# A Comparison of Image Statistics of Peacock Jumping Spider Colour Patterns and Natural Scenes

**DOI:** 10.1002/ece3.71363

**Published:** 2025-05-23

**Authors:** Marie‐Christin Hardenbicker, Joseph Schubert, Cynthia Tedore

**Affiliations:** ^1^ Institute for Animal Cell and Systems Biology University of Hamburg Hamburg Germany; ^2^ School of Life and Environmental Sciences University of Sydney Sydney New South Wales Australia

**Keywords:** Australian peacock spiders, efficient coding, Fourier transform, natural image statistics, processing bias, sexual signalling

## Abstract

The form of arbitrary sexual signals may be driven by the need to be detectable against the background or, alternatively, by selection for efficient processing by the nervous system. This latter alternative is a prediction of the sensory drive hypothesis extended to include efficient coding as a driver of the form of sexual signals. This hypothesis posits that animal visual systems are adapted to process the visual statistics of natural scenes, and that easily processed stimuli induce a sensation of pleasure in the viewer. In support of this, natural scene statistics have been found to be preferred not only by humans, but by the peacock spider *Maratus spicatus* . Here we test if male peacock spiders of the highly sexually dimorphic *Maratus* genus generally (a) evolve colour patterns with image statistics that contrast with the natural background or (b) exploit a potential processing bias by evolving colour patterns with visual statistics similar to those of natural scenes. We analyse and compare multispectral images of male and female spiders of 21 *Maratus* species and of natural scenes similar to the spiders' habitat. We find that the image statistics of male patterns diverge from those of natural scenes, whereas the statistics of female patterns do not. Our results support the idea that colour patterns evolve contrasting image statistics to increase conspicuousness and matching image statistics to be camouflaged. Any processing bias for natural scene image statistics in *Maratus* thus appears to play little role in the evolution of their sexual signals.

## Graphical abstract

What drives the evolution of colour patterns in peacock spiders: Standing out or processing bias? Hardenbicker et al. found that the image statistics of male colour patterns diverge from those of natural scenes, whereas the statistics of female patterns do not. It seems that when it comes to image statistics in *Maratus*, it is not about ease of processing but about being seen.

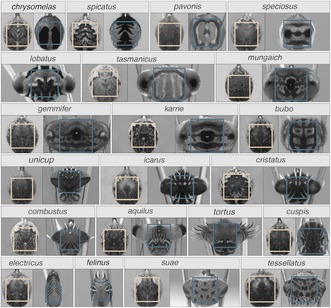

## 1 Introduction

Sexual selection pressure often leads to complex and elaborate courtship displays in the competing sex (Andersson 1994; Darwin 1871; Fisher 1930). Whereas some courtship signals are directly linked to information about the quality of a potential mate, others seem arbitrary, with their only benefit being the capacity to attract mating partners (Fisher 1930). How preferences that drive the evolution of arbitrary sexual signals evolve has long been subject to behavioural ecology research (e.g., Endler and Basolo 1998; Enquist and Arak 1993; Marler and Ryan 1997; Payne and Pagel 2001). Signal detection theory predicts that courtship signals should evolve to be conspicuous in front of a natural environment to increase efficacy (Endler 1992; Endler and Mappes 2017; Endler et al. 2005; Sibeaux et al. 2019; Wiley 2013). Conversely, the more recently introduced processing bias hypothesis, which couches efficient coding within a model of sensory drive, predicts that signals with statistics matching those of the natural environment are preferred because the receiver's sensory and nervous systems are tuned to process them more efficiently (Renoult and Mendelson 2019). It is likely that different mechanisms take effect for different traits and taxa, as well as at different stages of evolution; however, the relative frequency of different mechanisms is not well established.

For a signal to be effective in a mating context, it is crucial that it can be easily detected and discriminated by the receiver, that is, the choosy sex. This is particularly relevant when a signal is transmitted in a modality cluttered with extraneous stimuli. In the visual modality, extraneous stimuli could, for example, take the form of tangles of vegetation or variegated leaf litter. Animals can increase the probability of being detected either by exploiting pre‐existing biases, for example, by imitating properties of prey (e.g., Proctor 1991) or other food items like fruit (Rodd et al. 2002), or by expressing traits that contrast with the background and are hence more conspicuous. There are many examples where the need to be conspicuous, achieved by the level of contrast of a signal to the background, provides a good explanation for the form of sexual signals; that is, it is a good predictor for preference in a mating context. Males of the muscid fly *Lispe cana*, for example, display their bright iridescent faces against dark backgrounds to enhance signal contrast, which increases the attention they get from females, and with that, mating success (White et al. 2020). In the wolf spider *Schizocosa ocreata* , male forward‐facing secondary sexual traits used in visual signalling have colour values highly contrasting with the natural environment, whereas when viewed from above by a potential predator, the spiders' colours overlap with the background (Clark et al. 2011). The same is true for Aegean wall lizards (*Podarcis erhardii*), whose colourful visual signals are tuned to be conspicuous to conspecific vision while being less conspicuously perceived by avian predators in front of the same visual background (Marshall and Stevens 2014).

Conversely, framed within the context of sensory drive, the processing bias hypothesis predicts that signals that match the properties of the environment should be sexually selected for. The hypothesis assumes that perceptual systems are tuned to the statistical properties of their respective environment to reduce redundancy and process information with minimal cost. Visual systems can save metabolic energy by matching the arrangement of neurons and circuits to the statistical characteristics of the environment so that otherwise noisy visual input can be encoded to only use a small number of cells for transmission and processing in subsequent stages (Barlow 1961; Field 1987; Olshausen and Field 1996; Simoncelli 2003; Warrant 2016; Wehner 1987). It seems likely that animals have an innate preference or a pre‐existing bias for certain signal properties based on how efficiently they can be processed. A signal that matches the statistical properties that the system is tuned to should be processed using less energy, which potentially leads to a positive response and consequently elicits a preference (Dibot et al. 2023; Reber et al. 2004; Renoult and Mendelson 2019; Winkielman et al. 2003).

In addition to the more commonly studied properties of colour and brightness, visual signal properties are defined by spatial content; however, studies investigating the role of spatial content in visual courtship signals are scarce. Whereas colour and brightness in an image can be described by first‐order statistics using individual pixel values, summarising spatial information requires calculating the interaction of neighbouring pixels, that is, second‐order image statistics. Any given 2D signal can be approximated by the sum of 2D‐sinusoidal waves of particular amplitudes, spatial frequencies, and phases. Second‐order image statistics describe how pronounced brightness contrast is in a colour pattern at different spatial scales and orientations. A tool to measure relative brightness contrast across different spatial scales of an image is the Fourier transform. With it, we can decompose any image into the individual 2D‐sine waves that it is made of and measure their relative strength (= amplitude/brightness contrast) by transforming the image from the spatial domain into the frequency domain. Averaged over orientation, spatial frequency and amplitude typically have a linear relationship in a log–log plane, with low spatial frequencies usually having relatively high amplitudes and high frequencies having relatively low amplitudes. The slope of this linear relationship will hereafter be referred to as the spectral slope and is commonly used to describe the spatial frequency content of images (Cheney et al. 2014; Graham and Redies 2010; Hardenbicker and Tedore 2023; Hulse et al. 2020; Redies et al. 2007; Renoult and Mendelson 2019; Spehar et al. 2015).

Interestingly, Fourier analyses of images of natural scenes produce surprisingly uniform results when it comes to the spectral slope. In terrestrial habitats, amplitudes typically fall with increasing spatial frequency with a spectral slope of −1 (normally distributed, ranging from −1.6 to −0.7; Burton and Moorhead 1987; Field 1987; Pamplona et al. 2013; Párraga et al. 2000; Tolhurst et al. 1992; van der Schaaf and van Hateren 1996), which is typically attributed to scale‐invariance (Field 1987; Mandelbrot 1977; Ruderman 1994). This “natural spectral slope” has been associated with visual aesthetics in that humans seem to prefer images that possess this slope (Brichard et al. 2023; Isherwood et al. 2021; Spehar et al. 2015) and unconsciously recreate it in their artwork (Graham and Redies 2010; Redies 2007; Redies et al. 2007).

Comparatively little has been done to study a potential preference for the ‘natural spectral slope’ in other animals. In a recent analysis of colour patterns in different species of darters and their respective habitats, it was proposed that male colour patterns are adapted to be efficiently processed by matching the slope of their natural backgrounds (Hulse et al. 2020). The study found male slopes to be correlated but offset from habitat slopes such that their colour patterns were more dominated by lower spatial frequencies, whereas spectral slopes of female patterns showed no relation to habitat slopes. The correlation in males is suggestive that efficient coding could play a role in the spatial design of courtship patterns. However, the offset between male slopes and those of the background suggests a counteracting selective pressure for males to stand out from the background. A later study corroborated the idea that male darters are adapted to contrast with the background by comparing distances between darters and darter habitats in the feature space of individual layers of a convolutional artificial neural network (Hulse et al. 2022). Gram matrices (representative of image texture) of male darters plotted further away from darter habitats than those of females did, again suggesting that male colour patterns are adapted to stand out from the background. A recent study found evidence that a species of Australian peacock spider ( *Maratus spicatus* ) exhibits a preference for the ‘natural spectral slope’ of −1, which humans seem to prefer as well. However, females of this species have a pattern with a spectral slope closer to the natural slope than the slope of the male colour pattern, suggesting that this preference does not seem to be driving the evolution of male visual courtship display (Hardenbicker and Tedore 2023), although this might not be true for other species of this genus. The results given above are in line with results from earlier studies that investigated spectral slopes in the context of camouflage rather than in the context of sexual signalling. Cuckoo eggs, for instance, mimic the spectral slope of host eggs to avoid being identified as parasites (Stoddard and Stevens 2010). Cuttlefish alter their appearance so they match the slopes of the background (Barbosa et al. 2008), but when signalling, that is, when they intend to be seen, they change their pattern to contrast with the spectral slope of the background (Zylinski et al. 2011). Taken together, these findings suggest that colour pattern image statistics serve primarily as a means to decrease or increase colour pattern to background contrast. That said, studies of the image statistics of animal signals remain scarce, and more studies of a broader range of taxa are needed.

Australian peacock jumping spiders present an intriguing system to study the role of image statistics in the evolution of visual courtship displays. With currently about 100 described species, exhibiting a variety of elaborate courtship displays and intricate colour patterns, the *Maratus* genus is one of the most diverse groups in the animal kingdom when it comes to sexual signalling (Girard et al. 2021; Otto and Hill 2021). Peacock jumping spiders, like other salticids, have a highly developed visual system and many of their behaviours are visually guided (Land 1969). Accordingly, courtship displays, to a large extent, target the visual modality. A major part of the display is colourful ornaments on the abdomen, which males lift up during courtship and present towards females. In a detailed description and analysis of the complex courtship behaviour of *M. volans* , Girard et al. (2015, 2018) found that pattern contrast, the duration of visual display, but also to a lesser extent vibratory effort and vigour, influence mating success and other parameters that are linked to female choice. So far, Hardenbicker and Tedore's (2023) aforementioned study of *M. spicatus* is the only one that has looked at the spatial characteristics of *Maratus* colour patterns and what role they may play in the context of sexual selection. To determine whether the relationship between *M. spicatus* male and female slopes and those of natural scenes are representative of other *Maratus* species, in the present study, we expand our sampling to include 21 peacock spider species. We also analyse the slopes of ground microhabitats, since this is where *Maratus* are found. We aim to find out if what is true for *M. spicatus* is also true for other species of peacock jumping spiders; namely, whether (a) colour patterns of male peacock jumping spiders differ in their second‐order image statistics to the environment, whereas females have patterns with similar statistics, or (b) male slopes of other *Maratus* species are similar to the slope of natural scenes, which would suggest that a processing bias does, in fact, play a role in the evolution of colour patterns in the *Maratus* genus.

## 2 Methods

### 2.1 Multispectral Imaging

We photographed adult spiders of 21 different species of *Maratus* (for 18 species we photographed both sexes and for three species we only photographed males) with median sample sizes per species and sex of 5 (IQR: 1–5), depending on how many specimens could be found. Spiders were collected as adults between September and October 2019 in Western Australia, Australia (except *M. tasmanicus* , which was collected in Melbourne, Victoria, Australia). All individuals of *M. chrysomelas* and *M. spicatus* , as well as some individuals of *M. pavonis* , *M. mungaich* and *M. gemmifer* , were raised in the lab and photographed after they matured (for sample sizes and collection sites, see Table S1).

Before being photographed, each spider was killed overnight, while chilled in a refrigerator, by overanesthesia with CO_2_. The abdomen was then detached from the prosoma and positioned using insect pins such that it was oriented vertically as it would be during a male courtship display. For males with extendable flaps on the sides of the fan, these were held open in an extended position by additional insect pins. Spider abdomens were photographed in front of a 20% reflective 2‐in. fluorilon grey standard (Avian Technologies, New London, NH, USA), which reflects light evenly across the UV–VIS spectrum. We covered all surrounding surfaces with undyed brown paper with a reflectance spectrum similar to leaf litter. Adding to a naturalistic setup, specimens were illuminated by natural skylight with varying levels of cloud cover. To ensure there was no temporal variation in lighting across photos taken through different filters, we acquired multiple sets of the five images taken through each filter in sequence (i.e., images 1, 2, 3, 4, 5; 1, 2, 3, 4, 5; 1, 2, 3, 4, 5; etc.). Image sets with greater than 0.5% variation across three consecutive images taken through the same filter were discarded.

We used a multispectral camera with a customised set of filters. With this camera, we are able to mimic the salticid green channel, which peaks around 530 nm and is likely responsible for achromatic vision since it is the most densely packed photoreceptor class in the salticid retina (Blest et al. 1981, 1997; Blest and Price 1984; De Voe 1975; Land 1969; Nagata et al. 2012; Yamashita and Tateda 1976; Zurek et al. 2015). Spiders were photographed using three bird‐based filters (U, M and L) and two additional filters, previously described in Glenszczyk et al. (2021). From these filters, we calculated computational filters by taking the weighted sum of the existing set of five filters to generate new spectral sensitivities that matched the spectral sensitivity of the salticid green receptor (Blest et al. 1981; De Voe 1975; Glenszczyk et al. 2021; Yamashita and Tateda 1976; Zurek et al. 2015; for fits of computational sensitivity curves, see Figure S1a; the computational filter technique is further described in Tedore and Nilsson 2021; Glenszczyk et al. 2021; Tedore 2024). Since peacock spiders are very small, we added varying numbers and lengths of extension tubes (Kenko Extension Tube Set DG, Kenko Tokina Co. Ltd., Tokyo, Japan) between the 60 mm lens and the filter wheel of the camera according to the size of the respective species, such that the abdomen filled a large portion of the frame. Each extension tube allows focus at only a single distance from the camera lens. This setup enabled us to later measure the camera's field of view in millimetres for each extension, which was important for standardising the range of frequencies analysed in each image and for determining whether the analysed frequencies likely corresponded to those the spiders are capable of resolving (see Section 2.2).

Natural scenes photos were taken from the existing publicly available project dataset entitled ‘Natural Scenes through UVS and VS bird eyes: 6‐filter set’, available on multispectral.tedore.net (Tedore and Nilsson 2019, 2021; Tedore et al. 2022). Since peacock jumping spiders live on the ground, we decided to limit our analysis to 41 ground shots. Images in which the entire depth of field was not in focus were excluded from analysis. The scenes we chose are dominated by leaf litter and little sticks, which is what the typical habitat of the spiders consists of. Photos were taken at different locations in either Skåne, Sweden or Queensland, Australia and categorised into five different habitat types (for detailed information on each image, see Table S2). As described above, we converted the photos to images representative of the salticid green receptor by creating computational filters from the existing real filters in the camera (for fits of computational sensitivity curves, see Figure S1b).

Before converting the original, real filter images to computational filter images, we subtracted dark noise from all pixels, which we calculated from columns of the camera sensor that do not receive any light. Although we adjusted exposures to minimise the occurrence of over‐ and underexposed pixels, there were often a few isolated pixels, and in 5 of 145 images, 1–2 small clusters of pixels that were either over‐ or underexposed. After converting them to computational filters, we recalculated the values of these pixels using the mean of the surrounding adjacent pixels (excluding adjacent over‐ and underexposed pixels). After cropping images to a square for analysis (see below), only one image contained clusters of > 1 such pixel: one cluster of two and one cluster of eight overexposed pixels in a male specimen of *M. icarus* .

To simulate the adaptation of the eye and brain to the spectral distribution of light in a scene, we normalised each pixel value by the mean pixel value of the grey standard (for images of spider abdomens) or the mean pixel value of the image (for images of natural scenes). Following Naka and Rushton (1966), pixel values P were then converted to non‐linear receptor excitation values E,

(1) $$E=\frac{P}{P+1}$$

### 2.2 Calculation of Spectral Slope

**FIGURE 1:**
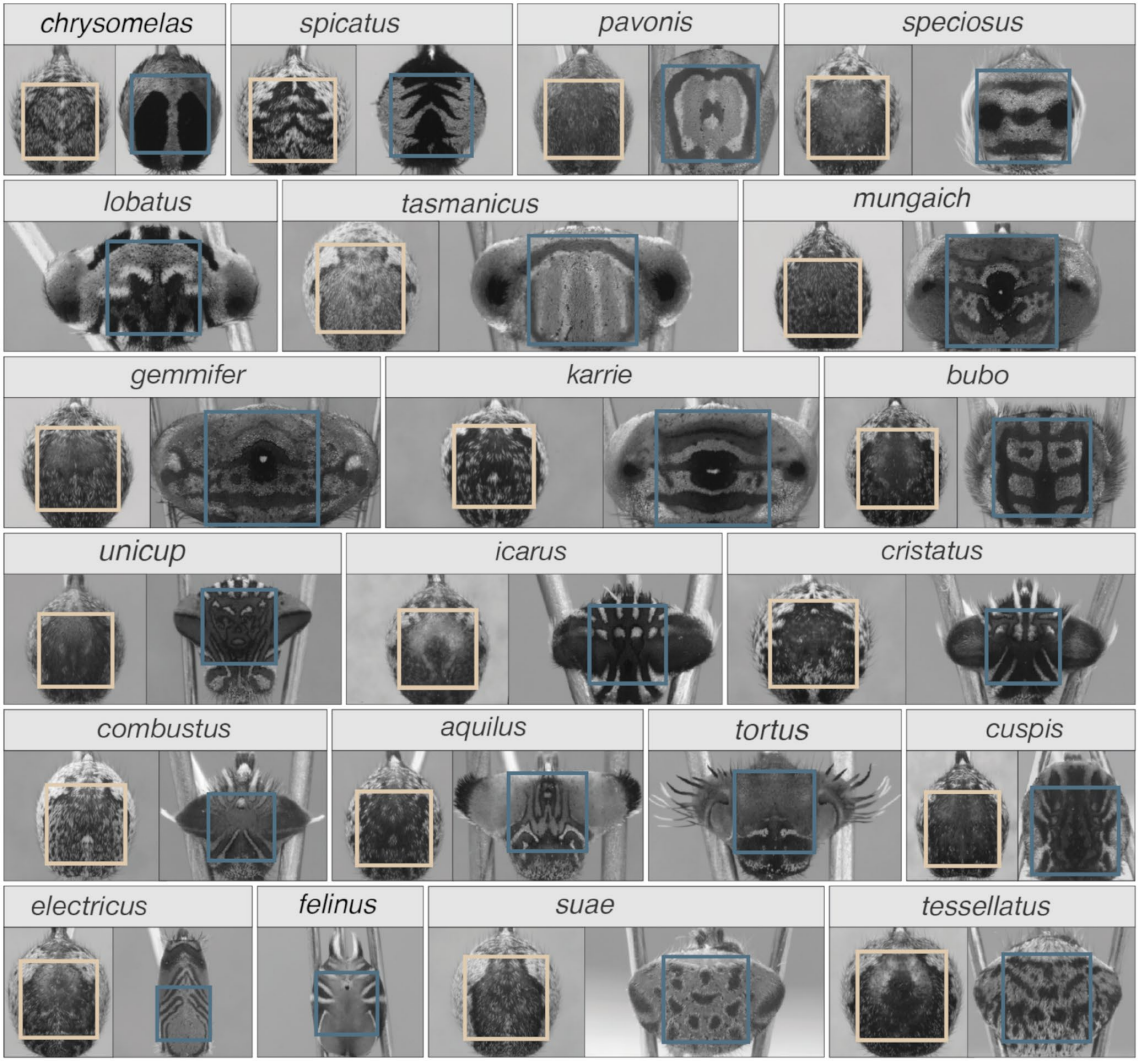
Computational filter images (matching the spectral sensitivity of the salticid green receptor that peaks at 530 nm) of the abdomens of male and female *Maratus* species (one example per species and sex). Squares indicate the area that was cropped from the image (yellow for females and blue for males) and used in the analysis to calculate the spectral slope.

To calculate the spectral slope, we transformed the images from the space domain to the frequency domain using a two‐dimensional fast Fourier transform. This analysis can only be applied to square images, so we cropped images of natural scenes from their original size of 1036 px × 1392 px to a square of 1036 px × 1036 px from the centre of the image. For images of spider abdomens, we extracted the largest square that could fit within the respective spider abdomen using a custom MATLAB script, resulting in images with an average size of 391 px × 391 px (±SD: 10 px; Figure 1). A cosine taper with a spatial frequency of four, tapered out to the mean pixel value of the respective image, was applied to the cropped images to avoid artificial edge effects. From the frequency domain, we calculated the spectral slope for each cropped image by fitting a line (using the least squares method) to the rotationally averaged amplitudes across spatial frequency in a log–log plane (Figures S2–S4). Regression models explained a substantial amount of variance, with *R* ^2^‐values for natural scene data ranging from 0.91 to 0.95 (mean: 0.93 ± SD: 0.01) and for spider data from 0.60 to 0.90 (mean: 0.81 ± SD: 0.06). To prevent aliasing and to exclude the cosine taper, we only considered frequencies ranging from a minimum of five cycles per cropped image to the dimension of each cropped image in pixels divided by 10. For the spider abdomen images, it was important to maintain consistency in the range of spatial scales considered between males and females of the same species because it is unknown whether their spectral slopes are scale‐invariant. Therefore, we further restricted the range of frequencies analysed in the spider images to be consistent across all individuals of the same species. To achieve this, we converted the minimum frequency analysed in each image to units of cycles per millimetre. We then identified the lowest such value across all individuals of a given species and excluded frequencies that fell below this range. We did this on a species‐by‐species basis rather than across the entire data set because substantial size variation across species would have caused visually prominent low frequencies to be excluded from larger species. Since the maximum spatial frequency analysed was calculated relative to the size of the crop (dimension in pixels divided by 10), the resulting maximum spatial frequency in units of cycles per mm was consistent for images photographed with the same extension tube. Two males of *M. chrysomelas* and five males of *M. spicatus* were photographed using 124 and 136 mm extension tubes, respectively, while all remaining images were taken through a 68 mm extension tube. We calculated the maximum frequency in units of cycles per millimetre for images taken with the shortest extension and adjusted the maximum spatial frequency in cycles per image of spiders photographed with larger extension tubes accordingly. This frequency *f*_max_ in units of cycles per millimetre was calculated as:

(2) $$f_{\max}=\frac{1392\;\mathrm{px}}{\mathrm{FOV}\times 10}$$

where FOV is the horizontal field of view of the camera with the 68 mm extension (5.7 mm) and 1392 is the original width of the image in pixels. This gave a value of 24.4 cycles/mm. To assess whether the spiders were likely able to resolve this spatial frequency, we estimated the spiders' highest possible spatial resolution in cycles/mm *f*_s_ by:

(3) $$f_{s}=\frac{1\;\mathrm{mm}}{\left(4d\tan\left(\frac{0.04^{{}^{\circ}}}{2}\right)\right)}$$

where *d* denotes the distance during courtship in mm and 0.04° is the inter‐receptor angle of the salticid *Portia fimbriata* (Williams and McIntyre 1980). *Maratus* males display at varying distances depending on the species, but during the majority of the courtship part that includes lifting and displaying the abdomen, males are quite close to the females (between 5 and 50 mm; M‐CH, personal observation). *M. spicatus* , for example, first lift their abdomens at an average distance of 27 mm before moving closer towards the female (Hardenbicker and Tedore 2023). Assuming the spiders' vision is in sharp focus at this distance, the spiders would be able to resolve a maximum of 26.5 cycles/mm, which is slightly higher than the maximum spatial frequency analysed (i.e., 24.4 cycles/mm). Although *Maratus*' small size compared to *P. fimbriata* makes this an optimistic upper limit for the details *Maratus* can resolve, peacock spiders' intricate visual courtship displays at short distances from the female suggest that they may indeed have relatively high spatial acuities at close distances.

Finally, in calculating the spectral slope, to account for the log scaled *x*‐axis, where high frequencies are more closely spaced and therefore have more weight, we averaged the mean amplitude of frequencies used for calculating the slope in nine evenly spaced bins.

### 2.3 Statistical Analysis

We fitted a Bayesian linear mixed‐effects model with two submodels using the ‘brm’ function from the ‘brms’ R package, version 2.20.1 (Bürkner 2017). Fitting the two submodels jointly within a multivariate framework enabled us to directly compare posterior estimates while accounting for shared variance and potential confounding factors.

In the first submodel, ‘spectral slope’ as a continuous response variable was predicted by slopes measured for male and female *Maratus*, using ‘sex’ as predictor variable. For this analysis, we only included species with data for both sexes (*N* = 137; for sample sizes per species and sex, see Table S1). We fitted species (18 levels) as a hierarchical grouping term to account for repeated observations per species, but we allowed species to vary by sex (model formula: spectral slope ~ sex + (sex|species)). Since female abdomens were typically larger than males', the number of pixels making them up was also larger. This meant that although the same range of frequencies in units of cycles per millimetre was used between males and females of the same species, the number of discrete frequencies this range was broken into was typically larger in females. We therefore assessed whether the number of discrete spatial frequencies used in the analysis should be included alongside sex in the model. We conducted a pre‐model comparison using 10‐fold cross‐validation with stratified sampling by species. We compared several candidate models, including models with number of frequencies as a main effect, an interaction term, and varying random slopes by species. The model including only sex and a random slope by species showed nearly identical predictive performance to more complex models, and substantially outperformed models including number of frequencies alone. Given the strong correlation between the number of frequencies and sex, and no improvement in predictive power when including this parameter, we retained the simpler model using only sex as a fixed effect in the multivariate model (for full model comparison results, see Table S3; for a plot showing the relationship between number of frequencies and spectral slope, see Figure S5).

In a second sub‐model, the spectral slope was predicted by our natural scenes data with ‘region’ (Skåne/Queensland) as a predictor variable (*N* = 41; see Table S2). We initially considered including ‘habitat’ as an additional covariate, but due to its strong correlation with region and the substantial imbalance in sample sizes across habitat levels, its inclusion prevented the model from converging. We therefore excluded ‘habitat’ from the final analysis.

The multivariate model was implemented using the Hamiltonian Monte Carlo (HMC) algorithm as a Markov Chain Monte Carlo (MCMC) sampling method, specifically the No‐U‐Turn Sampler (NUTS). Since our response variables followed a Gaussian distribution and their relationship with the predictors was linear, we specified ‘family’ as Gaussian with an identity link function. We ran the model with four chains, each consisting of 5000 iterations and a warm‐up period of 2500 iterations. We set ‘adapt_delta’ to 0.999 with a maximum tree depth of 10 to avoid false positive divergences. Since we had no specific prior information, priors were set as weakly informative with the default ‘flat’ distributions for Gaussian models. The model successfully converged (Rhat values = 1.00) and the Effective Sample Sizes (ESS) were > 1000 for all parameters, indicating that estimates are reliable.

We report posterior estimates (ES) and standard deviations (SD), as well as 95% credible intervals (CrI). If the CrI of the effect size (Δ) does not cross zero, we interpret this as credible evidence for an effect of the predictor.

## 3 Results

**FIGURE 2:**
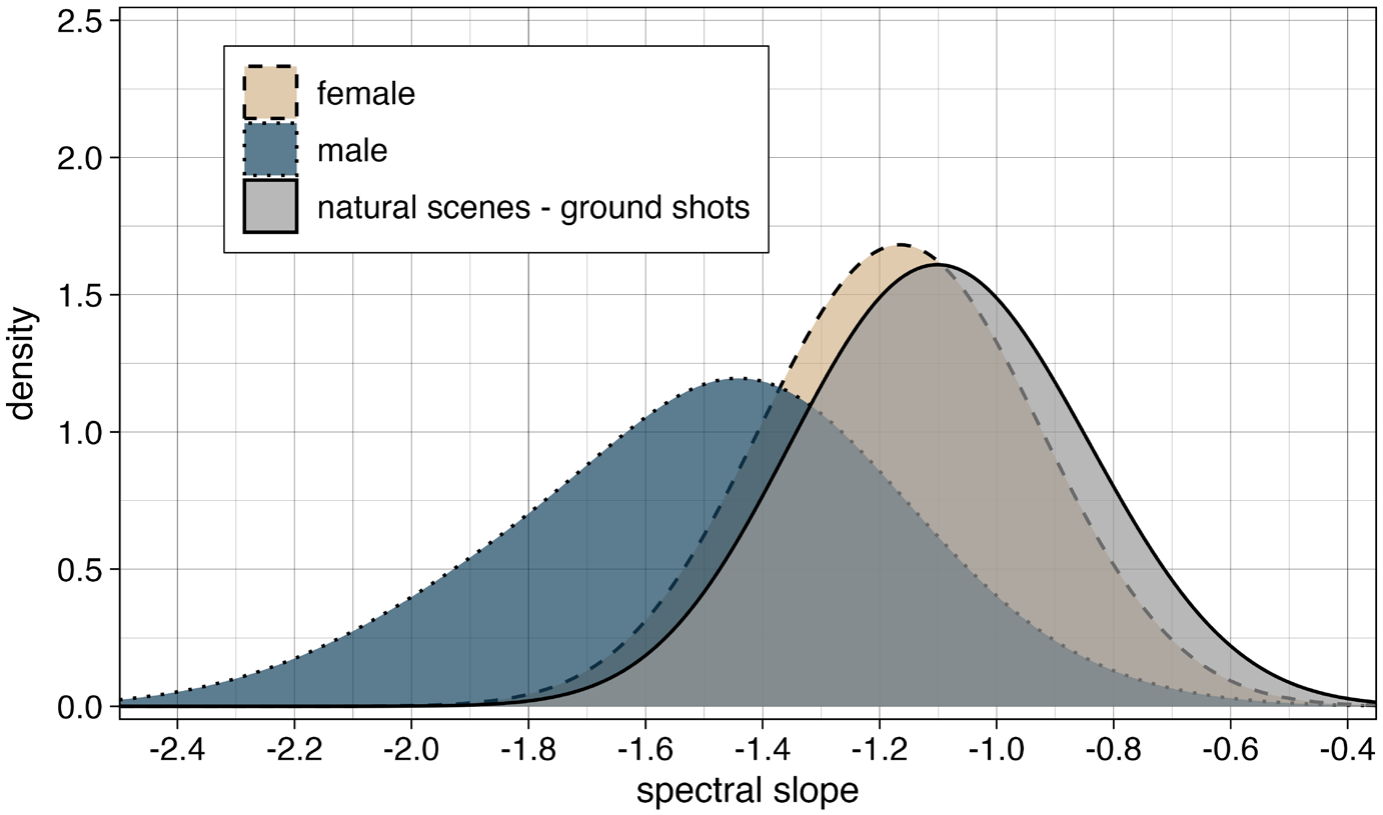
Distribution of spectral slopes of male and female *Maratus* from all species pooled (density plot based on raw data: SD of smoothing kernel = 0.2; females: *N*_species_ = 18, *N*_individuals_ = 49; males: *N*_species_ = 21, *N*_individuals_ = 94) compared to the distribution of slopes of natural scene images picturing the ground (*N* = 41).

We measured male and female spectral slopes as well as spectral slopes of images of natural scenes (only ground shots). Male slopes ranged from −2.15 to −0.79, with a mean slope of −1.5 ± SD: 0.28. Slopes of females and natural scenes were distributed very similarly, with females ranging from −1.43 to −0.91 (mean: −1.17 ± SD: 0.13) and natural scenes from −1.35 to −0.80 (mean: −1.09 ± SD: 0.14; Figure 2).

**FIGURE 3:**
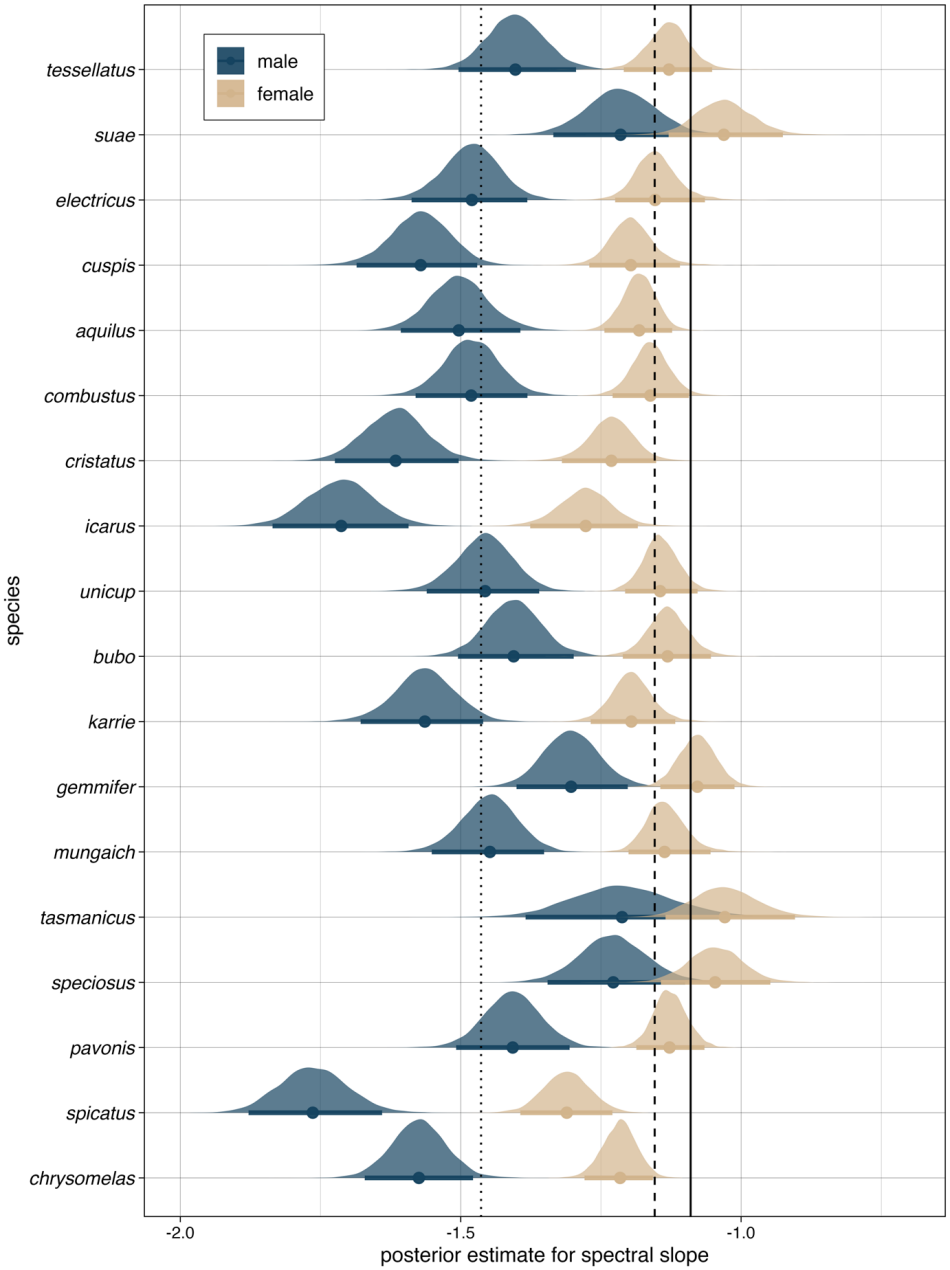
Distribution of posterior estimates for spectral slopes of male and female abdomens of 18 different *Maratus* species (Bayesian linear mixed effect model). Means per species and credible intervals are shown in a darker shade. Vertical lines represent means (dotted = males, dashed = females, solid = natural scenes; for boxplots of raw data, see Figure S6; for sample sizes per species, see Table S1).

In our model, the spectral slopes estimated for males differed substantially from those estimated for females, with Δ = −0.31 (SD: 0.05; CrI: −0.4 to −0.21; posterior estimates for each species: Figure 3). The slope for natural scene images, averaged over both regions, was estimated at −1.09 (SD: 0.02; 95% CrI: −1.14 to −1.05). Female slopes did not differ from spectral slopes of natural scene images (Δ = −0.06; SD: 0.04; CrI: −0.13 to 0.01), whereas male slopes did (Δ = −0.37; SD: 0.07; CrI: −0.5 to −0.24).

## 4 Discussion

Peacock jumping spiders are small (~2 to 6 mm) and live in cluttered environments like leaf litter and grass plants. Females usually hide in their surroundings and move very little, which makes them difficult to detect (M‐CH, JS and CT, personal observations). Since jumping spiders react to a moving stimulus by orienting towards it (Land 1971; Zurek et al. 2010), males can increase their chance of finding a female by triggering such a response. A conspicuous signal that catches female attention leads to the female orienting towards that signal. This movement then enables the male to locate the female (M‐CH, JS and CT, personal observations). Standing out is therefore of great importance to males, not only during courtship but also in the process of finding females, whereas female behaviour suggests that their main concern is blending in.

Our analysis of photos of male and female abdomens revealed a consistent pattern in 18 different species of *Maratus*, in that the spectral slopes of male ornaments diverge from the slopes of natural scenes, whereas female patterns have slopes close to the slope of natural scenes. These results would seem to indicate that male colour patterns evolve to have contrasting image statistics, which would increase their conspicuousness, and that female colour patterns evolve to have matching image statistics, making them better camouflaged in the natural environment. We did not find evidence for a processing bias influencing the form of *Maratus* sexual signals; that is, an influence of a preference for image statistics of natural scenes on the evolution of colour patterns relevant in courtship. The results of this study are in line with the findings of previous studies (Godfrey et al. 1987; Hardenbicker and Tedore 2023; Hulse et al. 2022; Zylinski et al. 2011) and provide more evidence for second‐order image statistics playing a role in colour pattern evolution.

Deviating from the spectral slope of the natural surroundings would seem to be an effective way to improve signal efficacy. However, there is evidence that visual systems are tuned to the image statistics of the natural environment and process visual input that possesses the same properties more efficiently, leading to a preference for such statistics (Hardenbicker and Tedore 2023; Redies 2007; Redies et al. 2007; Spehar et al. 2015). Yet, to date, no unequivocal evidence for sexual signals evolving to exploit this preference has been found. Still, the evolution of sexual signals could be influenced by the tuning of the visual system to the ‘natural spectral slope’, in that the range of spectral slopes that colour patterns evolve is limited by what can be perceived by the targeted visual system. Visual information might become distorted and not processable if it diverges too much from what the visual system is tuned to, making visual signals less likely to be seen. Hence, there could be a trade‐off between the need to stand out in front of the background and the risk of not being perceived because the signal deviates too far from what the eye is adapted to see. In our study system, male spectral slopes were always steeper than the ‘natural slope’/female slope, but usually, just outside the range of slopes, we measured for natural ground images, which could potentially be attributed to such a trade‐off.

Female slopes of some species (e.g., *M. spicatus* , *M. chrysomelas* , *M. icarus* and *M. cristatus* ) seem to diverge from the slope measured for natural scenes, although we have to account for low sample sizes per species, which makes it difficult to test for an effect of species on the spectral slope of male and female spiders. Regardless, sex differences across all species pooled together are strong, as is the similarity of female slopes to the natural slopes compared to male slopes. The variation that we observed among the spectral slopes of different species could potentially be explained by the spiders being adapted to the image statistics specific to their respective microhabitats. Since we did not measure the spectral slope of each species' microhabitat, we do not know whether there is significant variation among them. However, even though we only analysed natural scene images picturing the ground, we found a similar distribution of slopes to those found in previous studies for a variety of terrestrial habitats, supporting previous studies showing that the ‘natural slope’ of −1 is prevalent across different scales and habitats (Balboa and Grzywacz 2003; Field 1987; van der Schaaf and van Hateren 1996). Moreover, *M. spicatus* preferring random noise images that represented a slope of −1 (Hardenbicker and Tedore 2023) would make the most sense if this were the slope characteristic of their natural habitat. Female spectral slopes, when pooled, had a strikingly similar distribution to those of natural scenes, suggesting that variation in female slopes follows the natural variation of the ‘natural slope’ Male slopes were distributed much more broadly, which suggests that other factors besides the advantage of pattern‐background contrast may be driving this variation.

Our results show quite a clear pattern, but it is important to keep in mind that we are singling out one trait of a courtship display that consists of multiple traits that target more than one sensory modality. Courtship displays of Australian peacock jumping spiders are complex and involve other visual traits like colour and movement as well as vibrational and potentially chemical cues (Girard et al. 2015). There are several studies showing that multimodal signals can be more than the sum of their parts, due, for example, to temporal association between modalities (reviewed by Mitoyen et al. 2019). Consequently, caution is warranted when interpreting the evolutionary significance of single parts of a multimodal display, since it might be the composition of different traits that is selected for. With our study, we showed that male slopes deviate from the natural slope, but to confirm that contrasting spectral slopes actually increase female attention and with that the chance to mate, behavioural tests are needed. It is also worth noting that contrasting spectral slopes may not be selected for in their own right, but rather, could be a by‐product of other selective forces, such as the level of detail resolvable by different colour receptors in the spiders' eyes. All salticids measured so far have densely packed green receptors and sparsely distributed UV receptors (Blest et al. 1981; De Voe 1975; Land 1969), which means that to resolve a pattern with UV‐green contrast, the pattern would need to be made up of low spatial frequencies. Selection for UV‐green colour contrasts alone could therefore produce patterns dominated by lower spatial frequencies without a particular spectral slope being selected for per se.

In conclusion, the results of our study provide insight into the complex processes of colour pattern evolution in the *Maratus* genus. We identified a strong sexual dimorphism in spectral slope, showing that female slopes match the slope of the natural environment, whereas male slopes diverge and are more variable between species. With this study, we add to the large body of literature showing that conspicuousness achieved by colour patterns contrasting to the background is a major driver in the evolution of visual courtship signals (reviewed by Caves et al. 2024).

## Author Contributions

**Marie‐Christin Hardenbicker:** conceptualization (equal), data curation (equal), formal analysis (lead), methodology (equal), project administration (equal), resources (lead), software (equal), writing – original draft (lead). **Joseph Schubert:** resources (supporting), writing – review and editing (supporting). **Cynthia Tedore:** conceptualization (equal), data curation (equal), investigation (lead), methodology (equal), project administration (equal), resources (supporting), software (equal), supervision (lead), writing – review and editing (lead).

## Conflicts of Interest

The authors declare no conflicts of interest.

## Supporting information


Appendix S1

## Data Availability

The data that support the findings of this study will be openly available in Dryad at http://doi.org/10.5061/dryad.4f4qrfjnb.

## References

[ece371363-bib-0001] Andersson, M. B. 1994. Sexual Selection. Princeton University Press.

[ece371363-bib-0002] Balboa, R. M. , and N. M. Grzywacz . 2003. “Power Spectra and Distribution of Contrasts of Natural Images From Different Habitats.” Vision Research 43, no. 24: 2527–2537. 13129540 10.1016/s0042-6989(03)00471-1

[ece371363-bib-0003] Barbosa, A. , L. M. Mäthger , K. C. Buresch , et al. 2008. “Cuttlefish Camouflage: The Effects of Substrate Contrast and Size in Evoking Uniform, Mottle or Disruptive Body Patterns.” Vision Research 48, no. 10: 1242–1253. 18395241 10.1016/j.visres.2008.02.011

[ece371363-bib-0004] Barlow, H. B. 1961. “Possible Principles Underlying the Transformation of Sensory Messages.” Sensory Communication 1: 217–234.

[ece371363-bib-0005] Blest, A. D. , R. C. Hardie , P. McIntyre , and D. S. Williams . 1981. “The Spectral Sensitivities of Identified Receptors and the Function of Retinal Tiering in the Principal Eyes of a Jumping Spider.” Journal of Comparative Physiology A 145: 127–239.

[ece371363-bib-0006] Blest, A. D. , and G. D. Price . 1984. “Retinal Mosaics of the Principal Eyes of Some Jumping Spiders (Salticidae: Araneae): Adaptations for High Visual Acuity.” Protoplasma 120, no. 3: 172–184.

[ece371363-bib-0007] Blest, A. D. , C. Sigmund , and M. F. Land . 1997. “Retinal Mosaics of the Principal Eyes of Two Primitive Jumping Spiders, Yaginumanis and Lyssomanes: Clues to the Evolution of Salticid Vision.” Proceedings of the Royal Society of London. Series B: Biological Sciences 221, no. 1222: 111–125.

[ece371363-bib-0008] Brichard, Y. H. , M. Raymond , I. C. Cuthill , T. C. Mendelson , and J. P. Renoult . 2023. “From Natural to Sexual Selection: Revealing a Hidden Preference for Camouflage Patterns.” *bioRxiv*. 10.1101/2023.09.27.559753.

[ece371363-bib-0009] Bürkner, P.‐C. 2017. “brms: An R Package for Bayesian Multilevel Models Using Stan.” Journal of Statistical Software 80, no. 1: 1–28. 10.18637/jss.v080.i01.

[ece371363-bib-0010] Burton, G. J. , and I. R. Moorhead . 1987. “Color and Spatial Structure in Natural Scenes.” Applied Optics 26, no. 1: 157–170. 10.1364/AO.26.000157. 20454092

[ece371363-bib-0011] Caves, E. M. , A. L. Davis , S. Nowicki , and S. Johnsen . 2024. “Backgrounds and the Evolution of Visual Signals.” Trends in Ecology & Evolution 39, no. 2: 188–198. 37802667 10.1016/j.tree.2023.09.006

[ece371363-bib-0012] Cheney, K. L. , F. Cortesi , M. J. How , et al. 2014. “Conspicuous Visual Signals Do Not Coevolve With Increased Body Size in Marine Sea Slugs.” Journal of Evolutionary Biology 27, no. 4: 676–687. 24588922 10.1111/jeb.12348

[ece371363-bib-0013] Clark, D. L. , J. A. Roberts , M. Rector , and G. W. Uetz . 2011. “Spectral Reflectance and Communication in the Wolf Spider, *Schizocosa ocreata* (Hentz): Simultaneous Crypsis and Background Contrast in Visual Signals.” Behavioral Ecology and Sociobiology 65, no. 6: 1237–1247.

[ece371363-bib-0014] Darwin, C. 1871. The Descent of Man. John Murray.

[ece371363-bib-0015] De Voe, R. D. 1975. “Ultraviolet and Green Receptors in Principal Eyes of Jumping Spiders.” Journal of General Physiology 66, no. 2: 193–207. 1176947 10.1085/jgp.66.2.193 PMC2226199

[ece371363-bib-0016] Dibot, N. M. , S. Tieo , T. C. Mendelson , W. Puech , and J. P. Renoult . 2023. “Sparsity in an Artificial Neural Network Predicts Beauty: Towards a Model of Processing‐Based Aesthetics.” PLoS Computational Biology 19, no. 12: e1011703. 38048323 10.1371/journal.pcbi.1011703 PMC10721202

[ece371363-bib-0017] Endler, J. A. 1992. “Signals, Signal Conditions, and the Direction of Evolution.” American Naturalist 139: S125–S153.

[ece371363-bib-0018] Endler, J. A. , and A. L. Basolo . 1998. “Sensory Ecology, Receiver Biases and Sexual Selection.” Trends in Ecology & Evolution 13, no. 10: 415–420. 21238370 10.1016/s0169-5347(98)01471-2

[ece371363-bib-0019] Endler, J. A. , and J. Mappes . 2017. “The Current and Future State of Animal Coloration Research.” Philosophical Transactions of the Royal Society of London. Series B, Biological Sciences 372, no. 1724: 20160352. 28533467 10.1098/rstb.2016.0352 PMC5444071

[ece371363-bib-0020] Endler, J. A. , D. A. Westcott , J. R. Madden , and T. Robson . 2005. “Animal Visual Systems and the Evolution of Color Patterns: Sensory Processing Illuminates Signal Evolution.” Evolution 59, no. 8: 1795–1818. 16329248 10.1554/04-669.1

[ece371363-bib-0021] Enquist, M. , and A. Arak . 1993. “Selection of Exaggerated Male Traits by Female Aesthetic Senses.” Nature 361, no. 6411: 446–448. 8429883 10.1038/361446a0

[ece371363-bib-0022] Field, D. J. 1987. “Relations Between the Statistics of Natural Images and the Response Properties of Cortical Cells.” Journal of the Optical Society of America A: Optics and Image Science 4, no. 12: 2379–2394. 10.1364/JOSAA.4.002379. 3430225

[ece371363-bib-0023] Fisher, R. A. 1930. The Genetical Theory of Natural Selection. Clarendon Press.

[ece371363-bib-0024] Girard, M. B. , D. O. Elias , G. Azevedo , et al. 2021. “Phylogenomics of Peacock Spiders and Their Kin (Salticidae: *Maratus*), With Implications for the Evolution of Male Courtship Displays.” Biological Journal of the Linnean Society 132, no. 3: 471–494.

[ece371363-bib-0025] Girard, M. B. , D. O. Elias , and M. M. Kasumovic . 2015. “Female Preference for Multi‐Modal Courtship: Multiple Signals Are Important for Male Mating Success in Peacock Spiders.” Proceedings of the Royal Society B: Biological Sciences 282, no. 1820: 12–14. 10.1098/rspb.2015.2222 PMC4685782 26631566

[ece371363-bib-0026] Girard, M. B. , M. M. Kasumovic , and D. O. Elias . 2018. “The Role of Red Coloration and Song in Peacock Spider Courtship: Insights Into Complex Signaling Systems.” Behavioral Ecology 29: 1234–1244. 10.1093/beheco/ary128.

[ece371363-bib-0027] Glenszczyk, M. , D. Outomuro , M. Gregorič , et al. 2021. “The Jumping Spider *Saitis barbipes* Lacks a Red Photoreceptor to See Its Own Sexually Dimorphic Red Coloration.” Die Naturwissenschaften 109, no. 1: 6. 10.1007/s00114-021-01774-6. 34894274 PMC8665921

[ece371363-bib-0028] Godfrey, D. , J. N. Lythgoe , and D. A. Rumball . 1987. “Zebra Stripes and Tiger Stripes: The Spatial Frequency Distribution of the Pattern Compared to That of the Background Is Significant in Display and Crypsis.” Biological Journal of the Linnean Society 32, no. 4: 427–433.

[ece371363-bib-0029] Graham, D. J. , and C. Redies . 2010. “Statistical Regularities in Art: Relations With Visual Coding and Perception.” Vision Research 50, no. 16: 1503–1509. 20580643 10.1016/j.visres.2010.05.002

[ece371363-bib-0031] Hardenbicker, M.‐C. , and C. Tedore . 2023. “Peacock Spiders Prefer Image Statistics of Average Natural Scenes Over Those of Male Ornamentation.” Behavioral Ecology 34, no. 5: 719–728.

[ece371363-bib-0032] Hulse, S. V. , J. P. Renoult , and T. C. Mendelson . 2020. “Sexual Signaling Pattern Correlates With Habitat Pattern in Visually Ornamented Fishes.” Nature Communications 11, no. 1: 2561. 10.1038/s41467-020-16389-0 PMC7244530 32444815

[ece371363-bib-0033] Hulse, S. V. , J. P. Renoult , and T. C. Mendelson . 2022. “Using Deep Neural Networks to Model Similarity Between Visual Patterns: Application to Fish Sexual Signals.” Ecological Informatics 67: 101486.

[ece371363-bib-0034] Isherwood, Z. J. , C. W. G. Clifford , M. M. Schira , M. M. Roberts , and B. Spehar . 2021. “Nice and Slow: Measuring Sensitivity and Visual Preference Toward Naturalistic Stimuli Varying in Their Amplitude Spectra in Space and Time.” Vision Research 181: 47–60. 33578184 10.1016/j.visres.2021.01.001

[ece371363-bib-0035] Land, M. F. 1969. “Structure of the Retinae of the Principal Eyes of Jumping Spiders (Salticidae: Dendryphantinae) in Relation to Visual Optics.” Journal of Experimental Biology 51, no. 2: 443–470. 5351425 10.1242/jeb.51.2.443

[ece371363-bib-0036] Land, M. F. 1971. “Orientation by Jumping Spiders in the Absence of Visual Feedback.” Journal of Experimental Biology 54, no. 1: 119–139. 5549757 10.1242/jeb.54.1.119

[ece371363-bib-0037] Mandelbrot, B. B. 1977. Fractals: Form, Chance, and Dimension. W.H. Freeman.

[ece371363-bib-0038] Marler, C. A. , and M. J. Ryan . 1997. “Origin and Maintenance of a Female Mating Preference.” Evolution 51, no. 4: 1244–1248. 10.1111/j.1558-5646.1997.tb03971.x. 28565473

[ece371363-bib-0039] Marshall, K. L. A. , and M. Stevens . 2014. “Wall Lizards Display Conspicuous Signals to Conspecifics and Reduce Detection by Avian Predators.” Behavioral Ecology 25, no. 6: 1325–1337. 25419083 10.1093/beheco/aru126 PMC4235580

[ece371363-bib-0040] Mitoyen, C. , C. Quigley , and L. Fusani . 2019. “Evolution and Function of Multimodal Courtship Displays.” Ethology 125, no. 8: 503–515. 31341343 10.1111/eth.12882 PMC6618153

[ece371363-bib-0041] Nagata, T. , M. Koyanagi , H. Tsukamoto , et al. 2012. “Depth Perception From Image Defocus in a Jumping Spider.” Science 335, no. 6067: 469–471. 22282813 10.1126/science.1211667

[ece371363-bib-0042] Naka, K. I. , and W. A. H. Rushton . 1966. “S‐Potentials From Colour Units in the Retina of Fish (Cyprinidae).” Journal of Physiology 185: 536–555. 5918058 10.1113/jphysiol.1966.sp008001 PMC1395833

[ece371363-bib-0043] Olshausen, B. A. , and D. J. Field . 1996. “Emergence of Simple‐Cell Receptive Field Properties by Learning a Sparse Code for Natural Images.” Nature 381, no. 6583: 607–609. 8637596 10.1038/381607a0

[ece371363-bib-0044] Otto, J. C. , and D. E. Hill . 2021. “Catalogue of the Australian Peacock Spiders (Araneae: Salticidae: Euophryini: *Maratus*), Version 4.” Peckhamia 148.4: 1–35.

[ece371363-bib-0045] Pamplona, D. , J. Triesch , and C. A. Rothkopf . 2013. “Power Spectra of the Natural Input to the Visual System.” Vision Research 83: 66–75. 23458676 10.1016/j.visres.2013.01.011

[ece371363-bib-0046] Párraga, C. A. , T. Troscianko , and D. J. Tolhurst . 2000. “The Human Visual System Is Optimised for Processing the Spatial Information in Natural Visual Images.” Current Biology: CB 10, no. 1: 35–38. 10660301 10.1016/s0960-9822(99)00262-6

[ece371363-bib-0047] Payne, R. J. , and M. Pagel . 2001. “Inferring the Origins of State‐Dependent Courtship Traits.” American Naturalist 157, no. 1: 42–50. 10.1086/317007. 18707234

[ece371363-bib-0048] Proctor, H. C. 1991. “Courtship in the Water Mite *Neumania papillator* : Males Capitalize on Female Adaptations for Predation.” Animal Behaviour 42, no. 4: 589–598.

[ece371363-bib-0050] Reber, R. , N. Schwarz , and P. Winkielman . 2004. “Processing Fluency and Aesthetic Pleasure: Is Beauty in the Perceiver's Processing Experience?” Personality and Social Psychology Review 8, no. 4: 364–382. 10.1207/s15327957pspr0804_3. 15582859

[ece371363-bib-0051] Redies, C. 2007. “A Universal Model of Esthetic Perception Based on the Sensory Coding of Natural Stimuli.” Spatial Vision 21, no. 1–2: 97–117. 18073053 10.1163/156856807782753886

[ece371363-bib-0052] Redies, C. , J. Hasenstein , and J. Denzler . 2007. “Fractal‐Like Image Statistics in Visual Art: Similarity to Natural Scenes.” Spatial Vision 21, no. 1–2: 137–148. 18073055 10.1163/156856807782753921

[ece371363-bib-0053] Renoult, J. P. , and T. C. Mendelson . 2019. “Processing Bias: Extending Sensory Drive to Include Efficacy and Efficiency in Information Processing.” Proceedings. Biological Sciences/The Royal Society 286, no. 1900: 20190165. 10.1098/rspb.2019.0165. PMC6501678 30940061

[ece371363-bib-0054] Rodd, F. H. , K. A. Hughes , G. F. Grether , and C. T. Baril . 2002. “A Possible Non‐Sexual Origin of Mate Preference: Are Male Guppies Mimicking Fruit?” Proceedings. Biological Sciences/The Royal Society 269, no. 1490: 475–481. 10.1098/rspb.2001.1891. PMC1690917 11886639

[ece371363-bib-0055] Ruderman, D. L. 1994. “The Statistics of Natural Images.” Network: Computation in Neural Systems 5, no. 4: 517–548.

[ece371363-bib-0056] Sibeaux, A. , G. L. Cole , and J. A. Endler . 2019. “The Relative Importance of Local and Global Visual Contrast in Mate Choice.” Animal Behaviour 154: 143–159.

[ece371363-bib-0057] Simoncelli, E. P. 2003. “Vision and the Statistics of the Visual Environment.” Current Opinion in Neurobiology 13, no. 2: 144–149. 12744966 10.1016/s0959-4388(03)00047-3

[ece371363-bib-0058] Spehar, B. , S. Wong , S. van de Klundert , J. Lui , C. W. G. Clifford , and R. P. Taylor . 2015. “Beauty and the Beholder: The Role of Visual Sensitivity in Visual Preference.” Frontiers in Human Neuroscience 9: 514. 10.3389/fnhum.2015.00514. 26441611 PMC4585069

[ece371363-bib-0059] Stoddard, M. C. , and M. Stevens . 2010. “Pattern Mimicry of Host Eggs by the Common Cuckoo, as Seen Through a Bird's Eye.” Proceedings. Biological Sciences/The Royal Society 277, no. 1686: 1387–1393. 10.1098/rspb.2009.2018. PMC2871939 20053650

[ece371363-bib-0060] Tedore, C. 2024. “A Comparison of Photographic and Spectrometric Methods to Quantify the Colours Seen by Animal Eyes.” Methods in Ecology and Evolution 15, no. 1: 4–23. 10.1111/2041-210X.14255.

[ece371363-bib-0061] Tedore, C. , and D.‐E. Nilsson . 2019. “Avian UV Vision Enhances Leaf Surface Contrasts in Forest Environments.” Nature Communications 10, no. 1: 238. 10.1038/s41467-018-08142-5 PMC6342963 30670700

[ece371363-bib-0062] Tedore, C. , and D.‐E. Nilsson . 2021. “Ultraviolet Vision Aids the Detection of Nutrient‐Dense Non‐Signaling Plant Foods.” Vision Research 183: 16–29. 33639304 10.1016/j.visres.2021.01.009

[ece371363-bib-0063] Tedore, C. , K. Tedore , D. Westcott , C. Suttner , and D.‐E. Nilsson . 2022. “The Role of Detectability in the Evolution of Avian‐Dispersed Fruit Color.” Vision Research 196: 108046. 35381423 10.1016/j.visres.2022.108046

[ece371363-bib-0064] Tolhurst, D. J. , Y. Tadmor , and T. Chao . 1992. “Amplitude Spectra of Natural Images.” Ophthalmic & Physiological Optics: The Journal of the British College of Ophthalmic Opticians 12, no. 2: 229–232. 10.1111/j.1475-1313.1992.tb00296.x. 1408179

[ece371363-bib-0065] van der Schaaf, A. , and J. H. van Hateren . 1996. “Modelling the Power Spectra of Natural Images: Statistics and Information.” Vision Research 36, no. 17: 2759–2770. 8917763 10.1016/0042-6989(96)00002-8

[ece371363-bib-0066] Warrant, E. J. 2016. “Matched Filtering and the Ecology of Vision in Insects.” In The Ecology of Animal Senses: Matched Filters for Economical Sensing, edited by G. von der Emde and E. Warrant , 143–167. Springer.

[ece371363-bib-0067] Wehner, R. 1987. “‘Matched Filters’—Neural Models of the External World.” Journal of Comparative Physiology A 161, no. 4: 511–531. 10.1007/BF00603659.

[ece371363-bib-0068] White, T. E. , N. Vogel‐Ghibely , and N. J. Butterworth . 2020. “Flies Exploit Predictable Perspectives and Backgrounds to Enhance Iridescent Signal Salience and Mating Success.” American Naturalist 195, no. 4: 733–742. 10.1086/707584 32216666

[ece371363-bib-0069] Wiley, R. H. 2013. “Signal Detection, Noise, and the Evolution of Communication.” In Animal Communication and Noise, 7–30. Springer Berlin Heidelberg.

[ece371363-bib-0070] Williams, D. S. , and P. McIntyre . 1980. “The Principal Eyes of a Jumping Spider Have a Telephoto Component.” Nature 288, no. 5791: 578–580.

[ece371363-bib-0071] Winkielman, P. , N. Schwarz , T. A. Fazendeiro , and R. Reber . 2003. “The Hedonic Marking of Processing Fluency: Implications for Evaluative Judgment.” In The Psychology of Evaluation: Affective Processes in Cognition and Emotion, 189–217. Lawrence Erlbaum Associates Publishers.

[ece371363-bib-0072] Yamashita, S. , and H. Tateda . 1976. “Spectral Sensitivities of Jumping Spider Eyes.” Journal of Comparative Physiology 105, no. 1: 29–41.

[ece371363-bib-0073] Zurek, D. B. , T. W. Cronin , L. A. Taylor , K. Byrne , M. L. G. Sullivan , and N. I. Morehouse . 2015. “Spectral Filtering Enables Trichromatic Vision in Colorful Jumping Spiders.” Current Biology: CB 25, no. 10: R403–R404. 25989075 10.1016/j.cub.2015.03.033

[ece371363-bib-0074] Zurek, D. B. , A. J. Taylor , C. S. Evans , and X. J. Nelson . 2010. “The Role of the Anterior Lateral Eyes in the Vision‐Based Behaviour of Jumping Spiders.” Journal of Experimental Biology 213, no. Pt 14: 2372–2378. 10.1242/jeb.042382. 20581266

[ece371363-bib-0075] Zylinski, S. , M. J. How , D. Osorio , R. T. Hanlon , and N. J. Marshall . 2011. “To Be Seen or to Hide: Visual Characteristics of Body Patterns for Camouflage and Communication in the Australian Giant Cuttlefish *Sepia apama* .” American Naturalist 177, no. 5: 681–690. 10.1086/659626 21508613

