# Supplementary material for: A Comparison of Image Statistics of Peacock Jumping Spider Colour Patterns and Natural Scenes
Source: Ecol Evol. 2025 May 23;15(5):e71363. doi: 10.1002/ece3.71363 (PMC12101072; doi:10.1002/ece3.71363)
Supplement: Supplementary file 1 — Appendix S1 [file ECE3-15-e71363-s001.docx]

**Appendix**

**
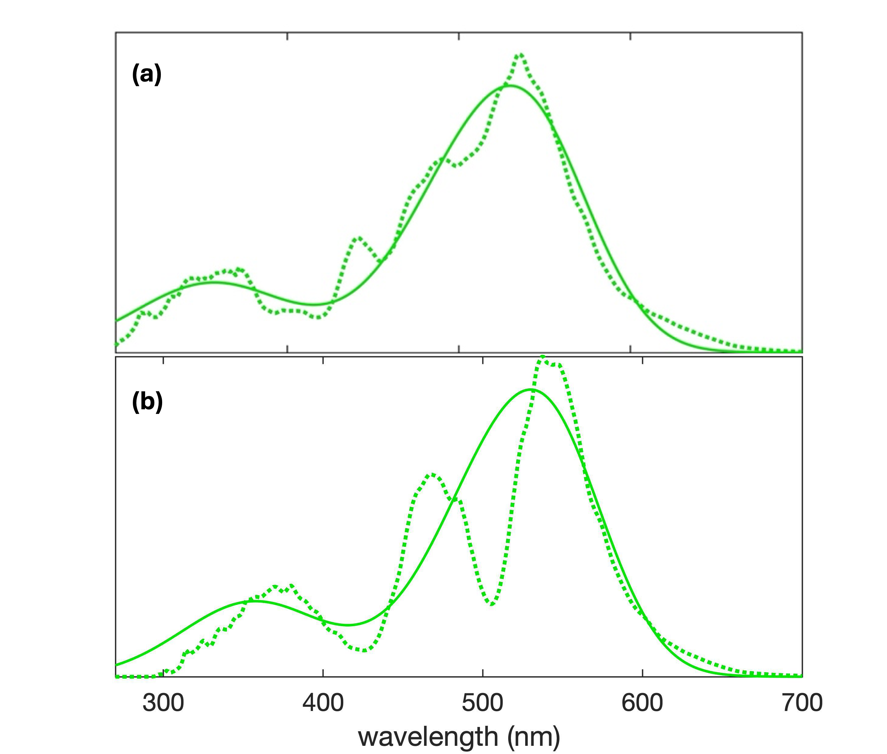
**

**Figure S1:** Normalized spectral sensitivity curve of computational filter (dotted) mimicking the spectral sensitivity of the salticid green receptor (solid) for **(a)** photos of spiders (5 filters set) and **(b)** natural scenes (6 filters set).

**Figure S2:** Rotationally averaged amplitude spectra of 41 images of natural scenes (grey lines). Frequencies five to the highest frequency divided by ten were averaged in nine evenly spaced bins and the spectral slope was calculated by linear regression. The green line represents the mean spectral slope of -1.09.

**Figure S3:** Rotationally averaged amplitude spectra of 49 female spiders (grey lines). Coloured lines show the mean spectral slope for each species, calculated by linear regression for frequencies five to the highest frequency divided by ten, averaged in nine evenly spaced bins.

**Figure S4:** Rotationally averaged amplitude spectra of a total of 94 male spiders (grey lines) grouped by species. Green lines represent mean spectral slope, calculated by linear regression for frequencies five to the highest frequency divided by ten, averaged in nine evenly spaced bins.

**Figure S5:** The number of discrete spatial frequencies used to calculate spectral slope plotted against the spectral slope per sex and species.

**
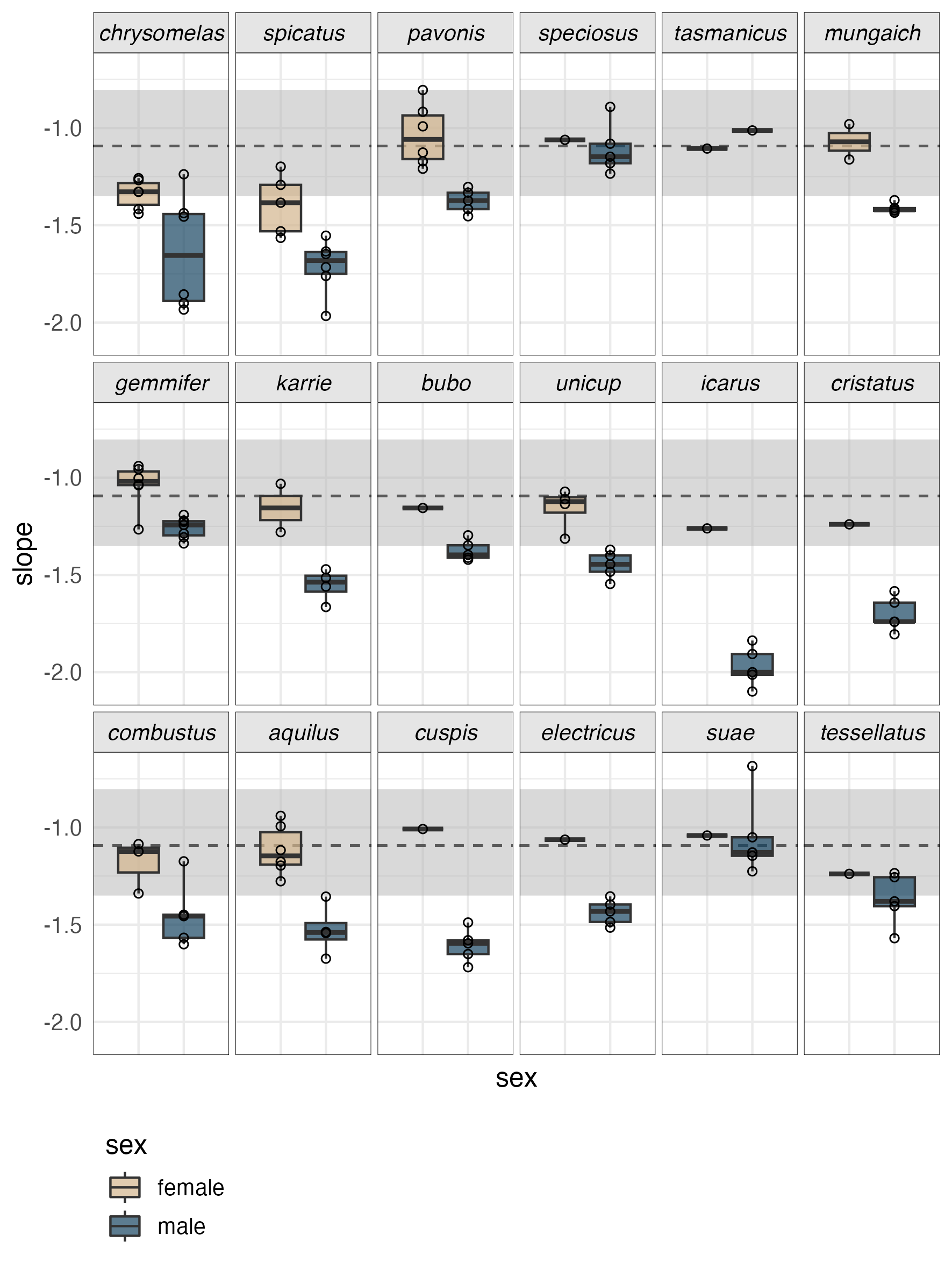
Figure S6:** Median spectral slopes of female and male abdomens of 18 different *Maratus* species. Black circles indicate the spectral slope of individual spiders’ abdomens. The grey area in each plot describes the range of spectral slopes that was measured in ground shots of natural scenes.

**Table S1:** Sample sizes and collection locations of *Maratus* specimens that were photographed for this study.

| **species** | **sample size** | | **generation** | **collection location** | | |
| --- | --- | --- | --- | --- | --- | --- |
|  | **male** | **female** |  |  | **latitude** | **longitude** |
| *chrysomelas* | 6 | 6 | F1, F2 | Mt. Dale (Helena national park), WA | 32°07'26.2"S | 116°17'32.8"E |
| *spicatus* | 6 | 5 | F1, F2 | Bow Bridge, WA | 34°58'05.6"S | 116°58'48.9"E |
|  |  |  |  | Leschenault Peninsula Conservation Park, WA | 33°14'07.6"S | 115°41'54.9"E |
| *pavonis* | 5 | 6 | parental, F1 | Leschenault Peninsula Conservation Park, WA | 33°14'07.6"S | 115°41'54.9"E |
|  |  |  |  | Mount Romance, WA | 34°51'10.2"S | 117°10'06.8"E |
|  |  |  |  | Quinninup, WA | 34°26'06.8"S | 116°15'18.9"E |
| *speciosus* | 5 | 1 | parental | The Maidens Walk, WA | 33°22'02.3"S | 115°36'55.5"E |
| *lobatus* | 1 | 0 | parental | Frankland River, WA | 34°22'31.5"S | 116°43'51.3"E |
| *tasmanicus* | 1 | 1 | parental | Melbourne, Vic | *NA* | *NA* |
| *mungaich* | 5 | 2 | parental, F1 | Mt. Dale (Helena national park), WA | 32°07'26.2"S | 116°17'32.8"E |
| *gemmifer* | 7 | 6 | parental, F1 | Baldivis, WA | 32°22'18.1"S | 115°49'34.2"E |
| *karrie* | 4 | 2 | parental | Manjimup Shire, WA | 34°35'22.5"S | 116°04'42.7"E |
| *bubo* | 5 | 1 | parental | North Walpole, WA | 34°51'39.7"S | 116°39'56.7"E |
| *unicup* | 5 | 4 | parental | Unicup Nature Reserve, WA | 34°22'31.1"S | 116°43'53.1"E |
| *icarus* | 5 | 1 | parental | North Walpole, WA | 34°54'43.6"S | 116°34'05.3"E |
| *cristatus* | 5 | 1 | parental | Bow Bridge, WA | 34°58'05.6"S | 116°58'48.9"E |
| *combustus* | 5 | 3 | parental | Mount Romance, WA | 34°51'09.9"S | 117°11'12.0"E |
| *aquilus* | 4 | 6 | parental | Mount Romance, WA | 34°51'06.5"S | 117°08'55.4"E |
| *tortus* | 1 | 0 | parental | North Walpole, WA | 34°47'05.9"S | 116°43'13.2"E |
| *cuspis* | 5 | 1 | parental | Mount Lindesay, WA | 34°50'11.6"S | 117°23'15.8"E |
|  |  |  |  | Trent, WA | 34°49'27.8"S | 116°58'12.8"E |
| *electricus* | 5 | 1 | parental | Lake Muir Nature Reserve, WA | 34°27'01.9"S | 116°41'01.3"E |
| *felinus* | 4 | 0 | parental | Lake Jasper, WA | 34°22'41.0"S | 115°39'28.3"E |
| *suae* | 5 | 1 | parental | Leschenault Peninsula Conservation Park, WA | 33°14'08.3"S | 115°41'54.4"E |
| *tessellatus* | 5 | 1 | parental | The Maidens Walk, WA | 33°22'02.0"S | 115°37'24.4"E |

**Table S2:** List of photos of natural scenes (ground shots) taken from data base.

| **id** | **region** | **location or nearest landmark** | **habitat** | **microhabitat** |
| --- | --- | --- | --- | --- |
| 154 | Skåne | Lomma Beach | temperate deciduous forest or rangeland | dead leaves mixed with grass, fruit |
| 173 | Skane | Skryllegården | temperate deciduous forest or rangeland | dead leaves mixed with grass |
| 187 | Skane | Dalby Norreskog | temperate deciduous forest or rangeland | dead leaves mixed with grass and moss |
| 188 | Skane | Dalby Norreskog | temperate deciduous forest or rangeland | grass and dead leaves |
| 203 | Queensland | Bellbird Clearing, Lamington National Park | subtropical rainforest | forbs, sticks but mostly dead leaves |
| 305 | Skane | Skryllegården | temperate deciduous forest or rangeland | grass and low shrubs |
| 418 | Skane | Ekologihus, Lund University Campus | temperate deciduous forest or rangeland | mostly dead leaves, some sticks |
| 429 | Queensland | Bellbird Lookout, Lamington National Park | subtropical rocky outcrop | dead leaves and sticks |
| 430 | Queensland | Atherton Tablelands Birdwatchers Cabin, Wondecla | tropical wet schlerophyll forest | branch, dead leaves and sticks |
| 431 | Queensland | Kuranda Birdwatchers Cabin | tropical rainforest | dead leaves, sticks and soil |
| 432 | Queensland | Kuranda Birdwatchers Cabin | tropical rainforest | dead leaves and sticks |
| 433 | Queensland | Kuranda Birdwatchers Cabin | tropical rainforest | dead leaves and sticks |
| 434 | Queensland | Kuranda Birdwatchers Cabin | tropical rainforest | dead leaves, sticks and soil |
| 435 | Queensland | Kuranda Birdwatchers Cabin | tropical rainforest | dead leaves and sticks |
| 436 | Queensland | Jindalba Boardwalk, Daintree Rainforest | tropical rainforest | dead leaves, branches, sticks and soil |
| 437 | Queensland | Jindalba Boardwalk, Daintree Rainforest | tropical rainforest | dead leaves and sticks |
| 438 | Queensland | Jindalba Boardwalk, Daintree Rainforest | tropical rainforest | dead leaves and sticks |
| 439 | Queensland | Daintree Discovery Center | tropical rainforest | dead leaves and sticks |
| 440 | Queensland | Marrdja Boardwalk, Daintree Rainforest | tropical rainforest | dead leaves, sticks and bark |
| 441 | Queensland | Jindalba Boardwalk, Daintree Rainforest | tropical rainforest | dead leaves, sticks and bark |
| 442 | Queensland | Jindalba Boardwalk, Daintree Rainforest | tropical rainforest | dead leaves, sticks and soil |
| 443 | Queensland | Jindalba Boardwalk, Daintree Rainforest | tropical rainforest | dead leaves, sticks and soil |
| 444 | Queensland | Marrdja Boardwalk, Daintree Rainforest | tropical rainforest | dead leaves, branches, sticks and soil |
| 445 | Queensland | Jindalba Boardwalk, Daintree Rainforest | tropical rainforest | dead leaves, branch |
| 446 | Queensland | Jindalba Boardwalk, Daintree Rainforest | tropical rainforest | dead leaves, branches, sticks and soil |
| 447 | Queensland | Tully Falls National Park | tropical rainforest | wet dead leaves |
| 448 | Queensland | Kuranda Birdwatchers Cabin | tropical rainforest | dead leaves and sticks |
| 480 | Queensland | Kuranda Birdwatchers Cabin | tropical rainforest | dead leaves, branches and sticks |
| 1251 | Queensland | Kuranda Birdwatchers Cabin | tropical rainforest | dead leaves and sticks |
| 1252 | Skane | Ekologihus, Lund University Campus | temperate deciduous forest or rangeland | mostly dead leaves, some sticks |
| 1253 | Skane | Ekologihus, Lund University Campus | temperate deciduous forest or rangeland | dead leaves, sticks and bark |
| 1255 | Queensland | Jindalba Boardwalk, Daintree Rainforest | tropical rainforest | mostly dead leaves, some sticks |
| 1256 | Queensland | Mount Lewis | tropical rainforest | dead leaves, branches, sticks and soil |
| 1257 | Skane | Ekologihus, Lund University Campus | temperate deciduous forest or rangeland | dead leaves and sticks |
| 1259 | Skane | Ekologihus, Lund University Campus | temperate deciduous forest or rangeland | dead leaves and sticks |
| 1260 | Skane | Ekologihus, Lund University Campus | temperate deciduous forest or rangeland | dead leaves and sticks |
| 1266 | Skane | Ekologihus, Lund University Campus | temperate deciduous forest or rangeland | dead leaves and sticks |
| 1267 | Skane | Ekologihus, Lund University Campus | temperate deciduous forest or rangeland | dead leaves and sticks |
| 1268 | Skane | Ekologihus, Lund University Campus | temperate deciduous forest or rangeland | dead leaves and sticks |
| 1269 | Skane | Ekologihus, Lund University Campus | temperate deciduous forest or rangeland | dead leaves and sticks |
| 1270 | Skane | Ekologihus, Lund University Campus | temperate deciduous forest or rangeland | dead leaves and sticks, some soil |

**Table S3**: Comparison of models predicting spectral slope of photos of *Maratus* abdomens, using stratified 10-fold cross-validation (stratified by species). Models are shown with fixed and random effect structures. elpd_diff indicates the difference in expected log predictive density relative to the best model (top row), and se_diff is the associated standard error.

| **Model formula** | **elpd_diff** | **se_diff** |
| --- | --- | --- |
| slope ~ sex * range + (sex * range \| species) | 0.0 | 0.0 |
| slope ~ sex * range + (sex + range \| species) | -2.4 | 1.4 |
| slope ~ sex + (sex \| species) | -2.7 | 2.9 |
| slope ~ sex + range + (sex \| species) | -3.7 | 2.5 |
| slope ~ sex + range + (sex + range \| species) | -4.8 | 2.6 |
| slope ~ range + (range \| species) | -46.7 | 8.3 |
| slope ~ range + (1 \| species) | -55.5 | 8.5 |
